# Fabrication of Uniform Nanoporous Oxide Layers on Long Cylindrical Zircaloy Tubes by Anodization Using Multi-Counter Electrodes

**DOI:** 10.1186/s11671-016-1774-1

**Published:** 2017-01-06

**Authors:** Yang Jeong Park, Jung Woo Kim, Ghafar Ali, Hyun Jin Kim, Yacine Addad, Sung Oh Cho

**Affiliations:** 1 Department of Nuclear and Quantum Engineering, Korea Advanced Institute of Science and Technology, 373-1 Guseong, Yuseong, Daejeon, 305-701 Republic of Korea; 2 Nanomaterials Research Group (NRG), Physics Division (PD), PINSTECH, Islamabad, 45650 Pakistan; 3 Department of Nuclear Engineering, Khalifa University of Science, Technology and Research, Abu Dhabi, 127788 United Arab Emirates

**Keywords:** Anodization, Electric field, Nanopore, Oxide layer, Simulation, Thickness, Zircaloy tube

## Abstract

We have presented a method to prepare a uniform anodic nanoporous oxide film on the surface of a cylindrical zircaloy (Zr) tube. The distribution of the electric field around the Zr tube determines the distribution of the thickness of the anodic nanoporous oxide film. The electric field generated when a cylindrical Zr tube is electrochemically anodized was simulated by using commercial code COMSOL. When four Pt wires were used as counter electrodes, a uniform electric field was achieved with minimal use of Pt. Based on the simulation results, a cylindrical Zr tube was anodized and the distribution of the thickness of the anodic nanoporous oxide layer was measured by FESEM. Also, mass production of uniform nanoporous anodic oxide films was possible by symmetrically arranging the zircaloy tubes and Pt wires.

**Electronic supplementary material:**

The online version of this article (doi:10.1186/s11671-016-1774-1) contains supplementary material, which is available to authorized users.

## Background

Electrochemical anodization has been widely used to produce oxide nanostructures on the surfaces of various metals [1–5] and alloys [6–9] due to simple and low-cost processing. It has been shown that the resulting morphology of the produced oxide nanostructure is affected by certain anodization parameters [1, 10, 11]. Nanoporous metal oxides are used for corrosion resistance [12, 13], decoration [14], templates for secondary nanomaterial fabrication [15, 16], and corrosion-proofing of a metal [17]. If a uniform and homogenous nanoporous oxide layer is created on a metal surface, water or moisture hardly contacts the underneath metal because the nanoporous oxide layer is free of gaps and cracks. Consequently, such a layer can act as a good protector against corrosion while a nanotubular oxide layer may not be quite corrosion-resistant.

To improve the safety of a nuclear power plant in the event of a severe accident, a zircaloy plate was anodized for the formation of a nanoporous zircaloy oxide layer. In fact, zircaloy is the most widely used nuclear fuel cladding material in nuclear reactors. If zircaloy comes into contact with high-temperature steam, the metal atoms in the zircaloy can react with oxygen molecules in the steam, leading to oxidation of the zircaloy. As a result, water molecules in the steam are dissociated and hydrogen gas is produced. If an excessive amount of hydrogen gas is produced, an explosion similar to the Fukushima nuclear power plant could happen [18]. If the surface of zircaloy is pre-oxidized through anodization, the oxide layer hinders further oxidation of the zircaloy cladding even in the steam environment, thereby preventing hydrogen production through water splitting. However, for this purpose, the oxide layer prepared on a zircaloy cladding should be of uniform and homogeneous. For application in nuclear reactors, nanoporous oxide structures of uniform thickness are to be fabricated on the surface of zircaloy cladding.

One important parameter of the process which strongly influences the thickness of the anodic oxide layer is the electric field [19, 20]. In the present study, the electric field is simulated considering electrostatics to produce a more uniform nanostructured oxide layer on the zircaloy tube surface. First, we developed an experimental setup based on the classical one dimension (1D). Then, a numerical model of the produced electric field is proposed to perform numerical simulations in a geometrical domain consisting of electrodes with boundary conditions. The numerical model is thus used to investigate the effect of several geometrical parameters, such as distribution of anodes and cathodes.

## Methods

Zircaloy tubes (KEPCO Nuclear Fuel Company. Ltd., 10φ mm, 0.7 mm thick) were used for the anodization experiments.The tubes were degreased by sonicating in acetone, isopropyl alcohol, and deionized water (DI) and dried with an air gun. Anodization was carried out using a cylindrical system with several platinum wires (0.2φ mm) as counter electrodes and a zircaloy tube as a working electrode. The distance between the anode and cathode was 20 mm. Ethylene glycol (95% purity, Junsei) containing ammonium fluoride (NH_4_F, Sigma-Aldrich Corporation, St. Louis, MO, USA) and DI water was used as an electrolyte. All the chemicals and materials were used in their as-received forms without any further purification. A direct current power supply was used for the electrochemical anodization. The anodization experiments were performed at 15 °C. After the experiments, the samples were rinsed with DI water and subsequently dried in air.

The thickness of the anodized film on the zircaloy (Zr) tube was examined by a field emission scanning electron microscope (FESEM, Nova230, FEI, USA) using a back-scattered electron (BSE) detector. For the cross-sectional measurement, the anodized sample was hot-mounted on purpose.

## Results and Discussion

Formation of oxide nanopores on a metal surface by anodization is generally explained by the competition between oxide layer formation and dissolution of the oxide layer. If zircaloy is anodized in an electrolyte containing aqueous F^−^ ions, the anodization processes are explained by the following reactions:

1-1 $$ \mathrm{Z}\mathrm{r}+2{\mathrm{OH}}^{-}\to {\mathrm{ZrO}}_2+2{\mathrm{H}}^{+}+4{\mathrm{e}}^{-}, $$

1-2 $$ 2{\mathrm{H}}^{+}+2{\mathrm{e}}^{-}\to {\mathrm{H}}_2, $$

2 $$ {\mathrm{ZrO}}_2+6{\mathrm{F}}^{-}+4{\mathrm{H}}^{+}\to {\left[{\mathrm{ZrF}}_6\right]}^{2-}+2{\mathrm{H}}_2\mathrm{O}, $$

3 $$ {\mathrm{Zr}}^{4+}+6{\mathrm{F}}^{-}\to {\left[{\mathrm{Zr}\mathrm{F}}_6\right]}^{2-}. $$

Since anions and cations are involved in the reactions, all the above reactions are strongly affected by an electric field generated between the zircaloy anode and cathode. In our previous study, the morphology of the oxide layer formed on the zircaloy surface is affected by anodization parameters such as anodization voltage, time, and water content in the electrolyte [21]. If the anodization parameters are properly adjusted, a zirconium oxide layer with complete nanoporous morphology could be produced on a flat zircaloy plate. However, in order to fabricate uniform nanoporous morphology on the surface, the electric field should be uniform and homogenous over the entire zircaloy surface. For a zircaloy plate, an almost uniform electric field can be achieved using a flat-shaped cathode. If a cylindrical-shaped zircaloy tube is anodized, a uniform electric field can be simply obtained over the entire surface of the zircaloy tube using a large tubular-shaped cathode, as shown in Additional file 1: Figure S1. A very large number of small holes in the platinum electrode are for smooth and uniform circulation of electrolytes. Distortion of the electric field coming from these holes is not significant when the distance between the platinum cathode and zircaloy cladding anode is sufficiently large, compared to the size of the holes. Generally, Pt is used as a cathode material in anodization to avoid unwanted side electrochemical reactions since Pt is very stable and reusable in electrolytes [22]. However, such a tubular-shaped Pt cathode is not effective because only one zircaloy tube is anodized using the tubular-shaped cathode and a high cost is required for the anodization process. In particular, if a very long (greater than a few meters) zircaloy tube is to be anodized, a large volume of expensive Pt is required, preventing the application of the simple anodization technique from being practical. Therefore, a more effective method is required.

**Fig. 1:**
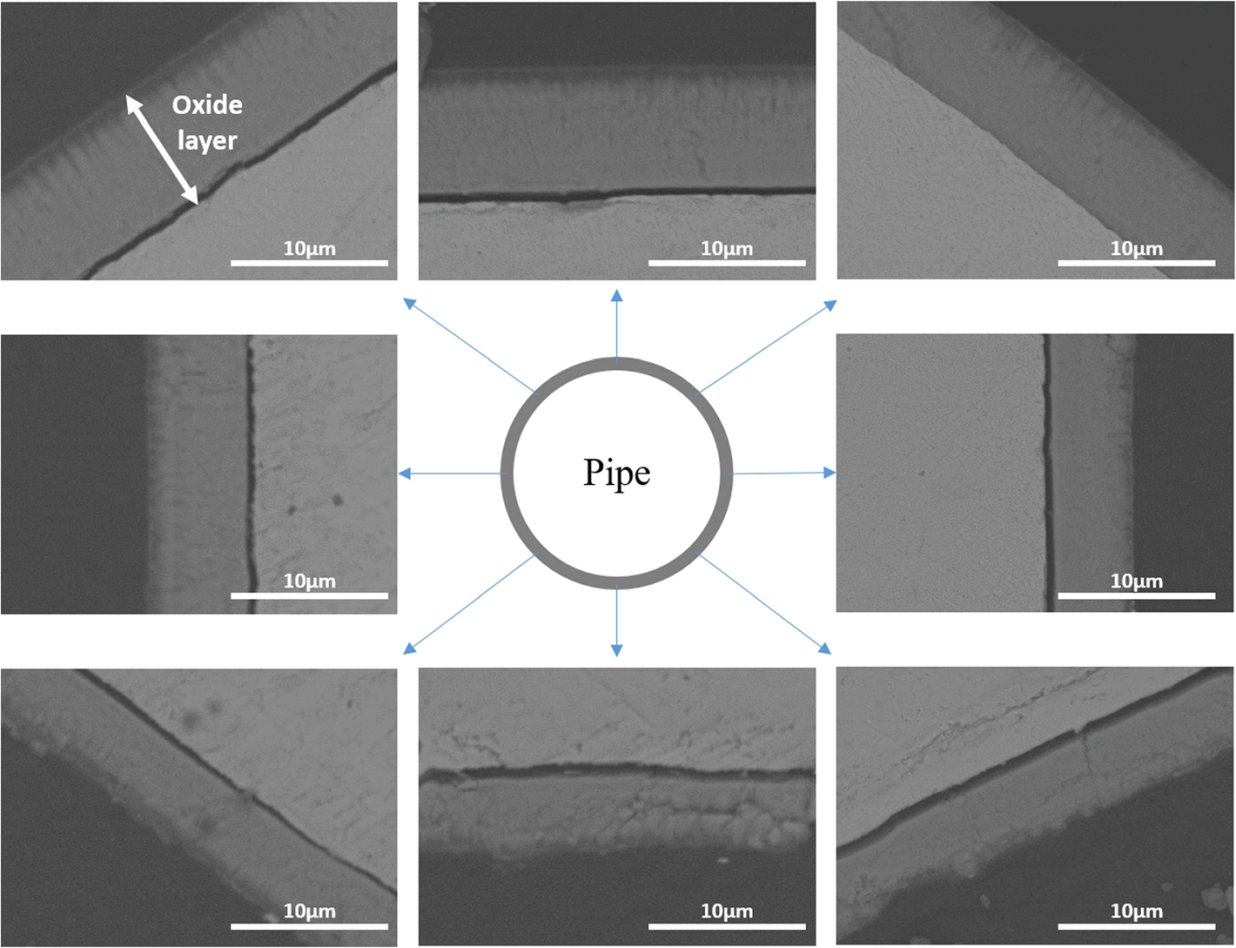
Cross-sectional BSE-FESEM images of the anodized zircaloy tube in ethylene glycol with 1 wt% H_2_O and 0.3 wt% NH_4_F for 5 min at the voltage of 90 V by using the one-wire Pt cathode

In this study, we adopted small Pt wires as a cathode during the anodization process. The Pt wire is less expensive compared to a Pt tube. Thus, a wire-shaped Pt cathode allows more practical application particularly if a very long zircaloy tube is to be anodized. First, in our anodization of the Zr tube, we used a single Pt wire as a cathode. The zircaloy tube was anodized in ethylene glycol with 1 wt% H_2_O and 0.3 wt% NH_4_F at 90 V for 5 min at 15 °C. The thickness of the oxide layer formed on the outer surface of the zircaloy tube was not uniform (Fig. 1). A thick oxide layer was created on the zircaloy tube surface closer to the cathode while a thin oxide layer was produced on the Zr tube surface away from the cathode (opposite side). The largest thickness of the oxide layer measured was 7.94 μm while the smallest thickness was 4.37 μm. This difference in the oxide layer thickness is attributed to the non-uniform electric field along the circular surface of the Zr tube.

The electric field distribution was only affected by the geometry of the electrode system. The electric field distribution according to the number of Pt wire cathodes was calculated by commercial code COMSOL. The calculated electric field distribution result and the oxide thickness distribution according to the number of Pt wire cathodes are shown in Additional file 1: Figure S2. It can be seen in Additional file 1: Figure S2 that the magnitude of the electric field is gradually decreased from the surface region facing the cathode to the opposite side of the Zr tube. As a result, it alters the anodization condition of each side of the zircaloy tube cladding. When a constant voltage of 90 V is applied between one Pt wire cathode (diameter 0.2 mm) and the zircaloy tube (diameter 10 mm), separated by 2 cm, the simulated result shows that the strongest electric field on the surface is 35.88 V/cm and the weakest field is 10.81 V/cm. It is interesting that an oxide layer is produced all over the outer surface of a zircaloy tube including the opposite side even though a single wire electrode is used. Although the difference between the electric field is three times, the difference between the thickest and thinnest is less than two times. This can be explained by the fact that the rate of oxide formation is determined by the pore etching rate, which is proportional to the anodization current [23]. Although the magnitude of the initial anodization current is proportional to the applied voltage, the former generally decreases with time due to the growth of the oxide layer on the surface of the materials [24]. In other words, a higher electric field results in a more rapid formation of the oxide layer on the surface of the materials initially; however, the anodization current eventually decreases rapidly and the oxide growth rate decreases. The average thickness of the created oxide layer is determined by the total charge flowing through the anode [6], which is calculated by integrating the anodization current with time.

**Fig. 2:**
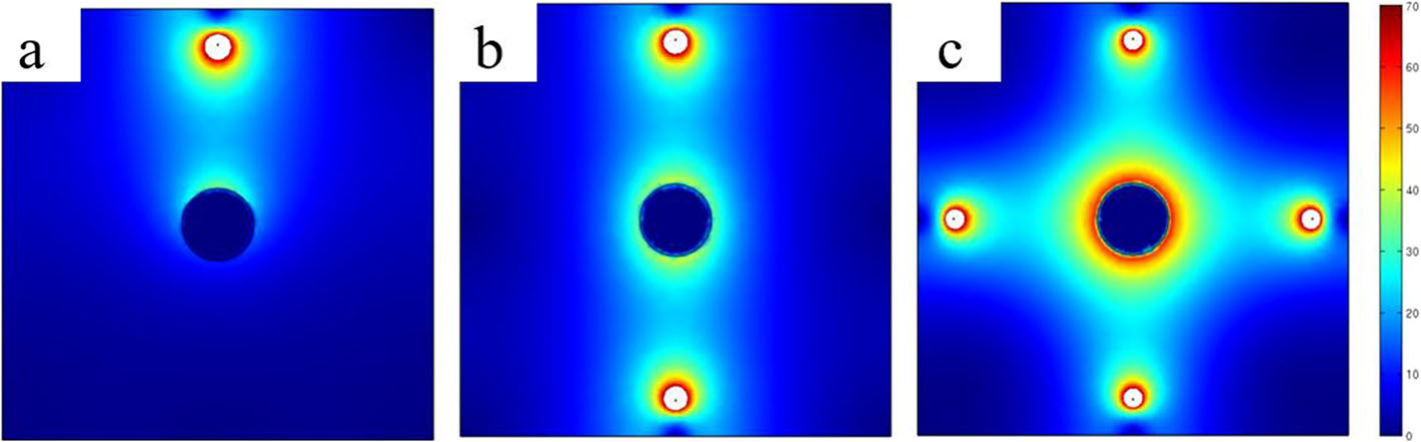
Simulation results of the electric field distribution of the **a** one-wire, **b** two-wire, and **c** four-wire Pt cathode system when 90 V of voltage is applied to the system

**Fig. 3:**
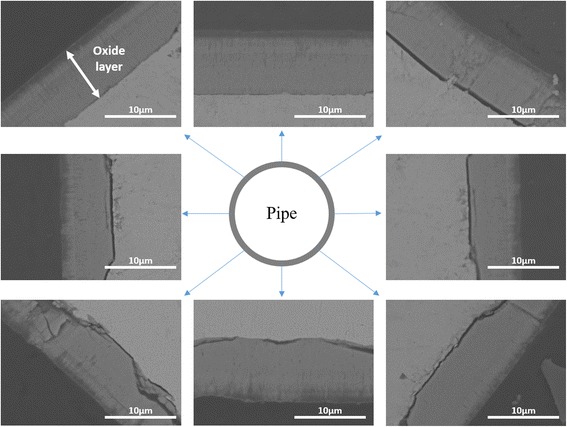
Cross-sectional BSE-FESEM images of anodized zircaloy tube in ethylene glycol with 1 wt% H_2_O and 0.3 wt% NH_4_F for 5 min at the voltage of 90 V by using the two-wire Pt cathode

**Fig. 4:**
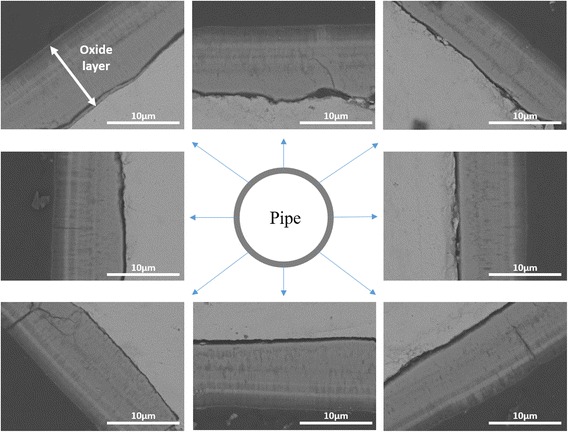
Cross-sectional BSE-FESEM images of anodized zircaloy tube in ethylene glycol with 1 wt% H_2_O and 0.3 wt% NH_4_F for 5 min at the voltage of 90 V by using the four-wire Pt cathode

The oxide layer with non-uniform thickness cannot be practically used for nuclear reactor application. The uniformity of the electric field distribution along the tube surface is simply defined as the ratio between minimum and maximum electric field strength and it can be improved by increasing the number of Pt wire cathodes. When two Pt wire cathodes were used, a better electric field distribution was achieved than a single Pt wire cathode (Fig. 2b). It is necessary to adjust electrodes in the right position for generation of uniform electric field throughout the metal surface. As a consequence, variation in the thickness of the oxide layer was reduced compared to a single wire electrode: the thickness of the oxide layer facing the Pt wire electrode was 9.01 μm (12, 6 o’clock position of Fig. 3) while the oxide layer formed at 90° had the thickness of 7.03 μm (3, 9 o’clock position of Fig. 3). Uniformity in the oxide layer thickness was much improved when 4 electrodes separated by 90° were used in the anodization. A slight electric field variation around the Zr tube has been found. According to the simulation/calculation, the electric field changes around the cylindrical surface are from 61.26 to 63.92 V/cm. The uniformity of the electric field distribution is around 0.96 and this is quite acceptable. Moreover, the thickness of the fabricated oxide layer was almost constant: the maximum and the minimum thickness were 10.6 and 9.22 μm, respectively. FESEM characterization reveals that a uniform and homogenous nanoporous oxide structure was produced on the entire outer surface of a zircaloy tube (Fig. 4) when four Pt wire cathodes were used.

**Fig. 5:**
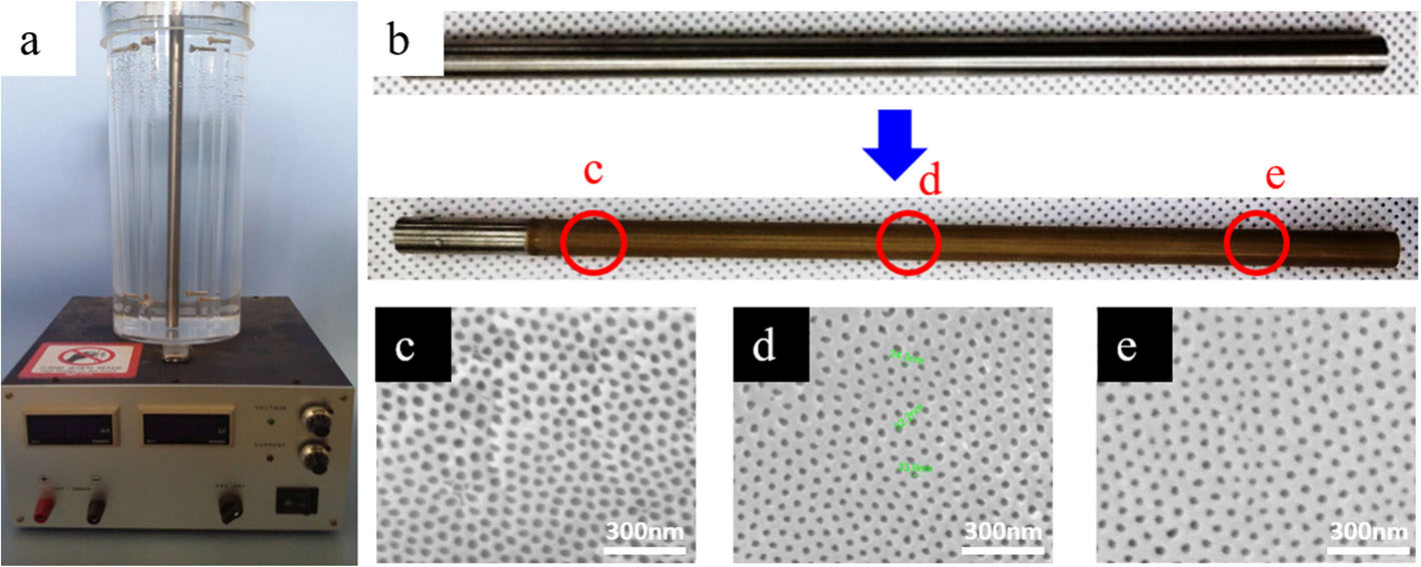
**a** Mechanical support system of the 30-cm-long zircaloy tube. **b** The anodized result of the tube. **c–e** FESEM images of nanoporous ZrO_2_ layers fabricated at different positions

**Fig. 6:**
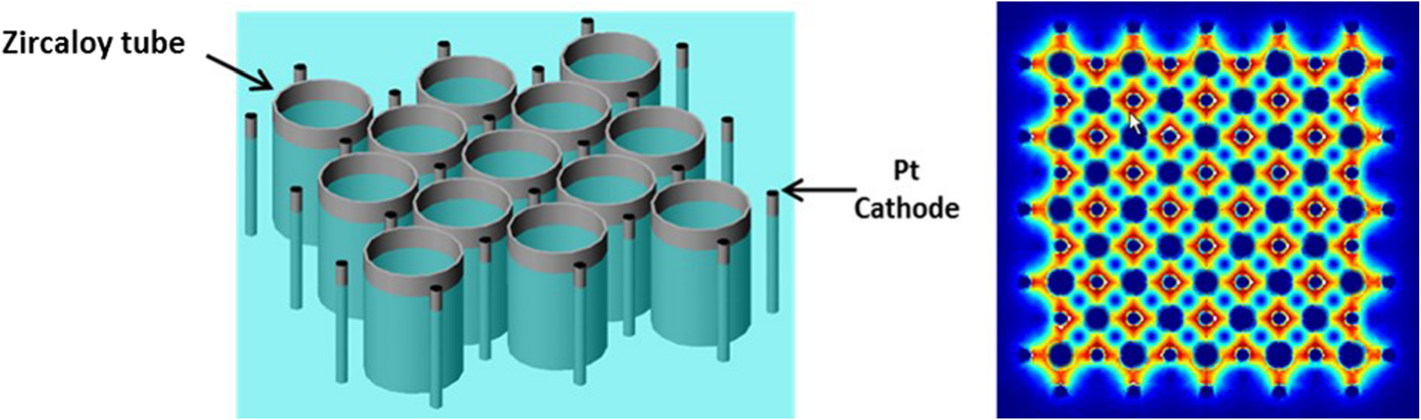
Schematic image of regularly and two-dimensionally arranged zircaloy tubes and wire electrodes with the calculation result of the electric field distribution of the arrangement

In addition, we used the four Pt wire cathodes for the anodization of long (30 cm) zircaloy tubes under the same anodization conditions. The Pt wires were fixed around the zircaloy tubes using a mechanical support as shown in the Fig. 5a. FESEM images show that nanoporous structures were uniformly created over the whole surface of the long zircaloy tube. The fabricated nanostructure has a honeycomb like hexagonal close-packed structure without any gaps unlike the nanotubular structure. And, the oxide layer thickness is around 10 μm, uniformly distributed along the entire surface. Another advantage of the four-wire cathode system is that multi-tubes can be simultaneously anodized using a two-dimensional arrangement of the wire electrodes. If zircaloy tubes are regularly and two-dimensionally arranged, empty spaces are naturally formed between the tubes and the wire electrodes can be installed in these empty spaces. This kind of arrangement is shown in the schematic diagram with the calculation result of the electric field distribution of the arrangement (Fig. 6). If the length of the wire electrodes is longer than the cladding pipe, a nanostructured oxide layer will be prepared on the whole surface of the cladding pipe at the same time. This geometry allows a cheap, quick, and effective route to the mass production of anodized zircaloy tubes.

## Conclusions

In the present work, the role of electric field strength was studied on the growth morphology of nanopores through electrochemical anodization using a cylindrical electrode system. A numerical model is developed to perform simulations on an electric field distribution of the cylindrical electrode system. The electric field distribution deviation is reduced when the number of Pt wire cathodes increases. An almost uniform electric field was obtained when four Pt wire cathodes were used in the anodization. FESEM results reveal that the thickness of the anodic oxide layer on the zircaloy tube increases with increase in electric field strength. Consequently, the zircaloy tube with a uniform anodic oxide layer can be fabricated by using the four-wire cathode system. We suggest that the proposed model can also be applied to other metals and alloys for the mass production of uniform anodization. Therefore, the zircaloy tubes with anodic metal oxides can exhibit enhanced performances for various applications.

## Additional File

Additional file 1: Figure S1. Tubular-shaped Pt cathode with a lot of small holes and an anodization system using the cathode. **Figure S2.** a) Oxide thickness distribution and b) electric field distribution according to the angular position of the Zr-Nb-Sn tube. The tube anodized in the one-, two-, three-, and four-wire system are marked as black, red, purple, and blue lines, respectively. (DOC 408 kb)

## Abbreviations

BSE: Back-scattered electron
FESEM: Field emission scanning electron microscope
