# Supplementary material for: Fabrication of Uniform Nanoporous Oxide Layers on Long Cylindrical Zircaloy Tubes by Anodization Using Multi-Counter Electrodes
Source: Nanoscale Res Lett. 2017 Jan 6;12:20. doi: 10.1186/s11671-016-1774-1 (PMC5216014; doi:10.1186/s11671-016-1774-1)
Supplement: Additional file 1: Figure S1. — Tubular-shaped Pt cathode with a lot of small holes and an anodization system using the cathode. Figure S2. a) Oxide thickness distribution and b) electric field distribution according to the angular position of the Zr-Nb-Sn tube. The tube anodized in the one-, two-, three-, and four-wire system are marked as black, red, purple, and blue lines, respectively. (DOC 408 kb) [file 11671_2016_1774_MOESM1_ESM.doc]

**Additional file 1**

**Fabrication of uniform nanoporous oxide layers on long cylindrical Zircaloy tubes by anodization using multi-counter electrodes**

Yang Jeong Park, Jung Woo Kim, Ghafar Ali, Hyun Jin Kim, Yacine Addad

and Sung Oh Cho[[1]](#footnote-2)*


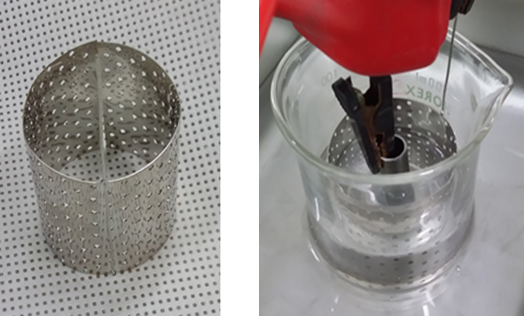


**Figure S1.** Tubular-shaped Pt cathode with lots of small holes and anodization system using the cathode.


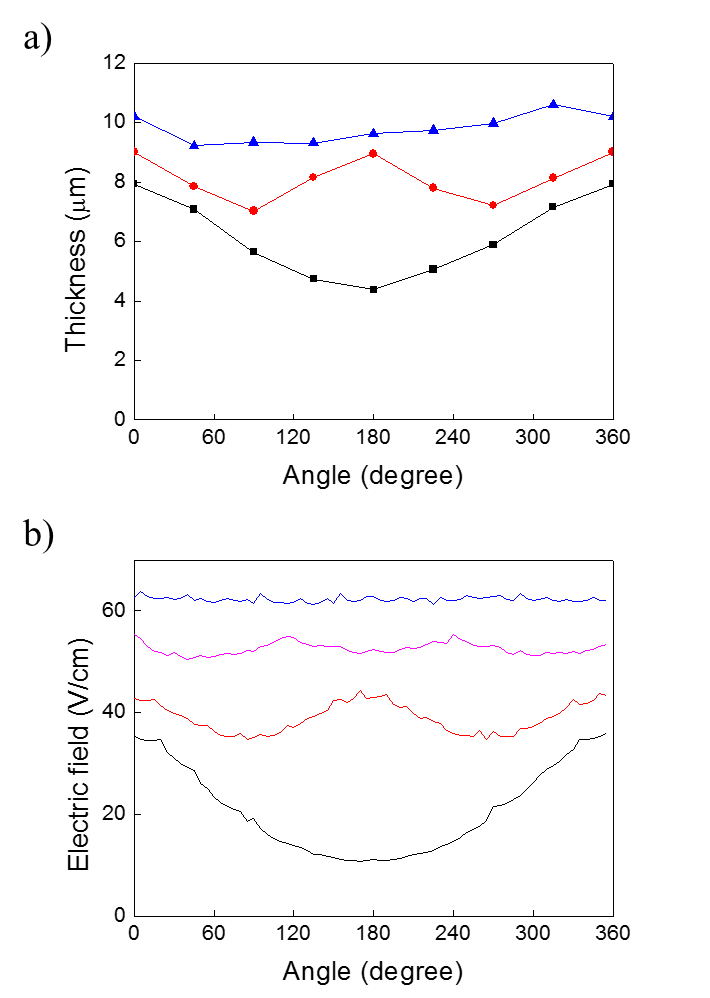


**Figure S2.** a) Oxide thickness distribution and b) electric field distribution according to the angular position of the Zr-Nb-Sn tube. The tube anodized in 1,2,3,4 wire system marked as black, red, purple, blue line, respectively.

1. ^^Corresponding Author (**S. O. Cho**); Tel.: +82 42 350 3823, Fax: +82 42 350 3810, E-mail: [socho@kaist.ac.kr](mailto:socho@kaist.ac.kr) [↑](#footnote-ref-2)
